# Development of a complex intervention to support parents of adolescents with chronic illness transferring from pediatrics to adult care (ParTNerSTEPs)

**DOI:** 10.1186/s12913-022-07888-5

**Published:** 2022-04-12

**Authors:** Ena Lindhart Thomsen, Bente Appel Esbensen, Signe Hanghøj, Helena Hansson, Kirsten Arntz Boisen

**Affiliations:** 1 Department of Paediatrics and Adolescent Medicine, Center of Adolescent Medicine, Copenhagen University Hospital – Rigshospitalet, Blegdamsvej 9, 2100 Copenhagen O, Denmark; 2 Copenhagen Center for Arthritis Research (COPECARE), Center of Rheumatology and Spine Disorders, Centre of Head and Orthopaedics, Copenhagen University Hospital – Rigshospitalet, Valdemar Hansens Vej 13-17, 2900 Glostrup, Denmark; 3 Department of Clinical Medicine, Faculty of Health and Medical Sciences, University of Copenhagen, Blegdamsvej 3B, 2200 Copenhagen, Denmark; 4 Department of Paediatric and Adolescent Medicine, Copenhagen University Hospital – Rigshospitalet, Blegdamsvej 60B, 2100 Copenhagen, Denmark

**Keywords:** Adolescent, Chronically ill, Complex intervention, Development, Parents, Participatory design, Transitional care

## Abstract

**Background:**

Transition from pediatric to adult care for adolescents with chronic illness is associated with outpatient non-attendance and low treatment adherence in adolescents, and with anxiety and concerns among parents. Recent studies have shown that parent involvement results in better transitions. The aim of this paper was to describe the development, through participatory design, of a comprehensive transfer program targeted to parents of adolescents with chronic illness.

**Methods:**

The study was based on the UK Medical Research Council’s (MRC) framework on developing and testing complex interventions. To increase the program’s feasibility and relevance, participatory design was chosen as the overall method. A collaboration group of parents, young people and health care professionals (HCP) were actively involved in the development of the program. The program was developed in three development stages, in accordance with the MRC framework: 1) identifying the evidence base, 2) identifying theory, and 3) modelling process and outcomes.

**Results:**

Together with the collaboration group, we developed a comprehensive transfer program targeting parents, by undertaking an iterative process, involving a literature review, individual interviews, workshops and online brainstorms. The program, called ParTNerSTEPs (Parents in Transition – a Nurse-led Support and Transfer Educational Program) comprised three components: 1) an informative website, 2) online educational events for parents, and 3) transfer consultations with providers from both pediatrics and adult care.

**Conclusions:**

The MRC framework was successfully applied to develop a comprehensive transfer program targeting parents of adolescents with chronic ilness. By incorporating the principles of participatory design in the development phase, we ensured that both parents’ and adolescents’ needs were represented and addressed in the program.

**Trial registration:**

ClinicalTrials.gov ID NCT04969328.

**Supplementary Information:**

The online version contains supplementary material available at 10.1186/s12913-022-07888-5.

## Background

The transition from pediatrics to adult care for adolescents with chronic illness is associated with medical complications, outpatient non-attendance and low treatment adherence [1–3]. Lately, it has become evident that one of the most important factors in a successful transition and transfer to adult care is appropriate parental involvement and that parents are feeling ready for their child to transfer [4–6]. The parents’ role is also highlighted in transition theories, e.g., the social-ecological model of adolescent and young adult readiness to transition (SMART) [7]. In that model, the parents’ ability to coach and support their child in gaining self-management skills is described as a facilitator of the transition process [8].

The parents’ role in their child’s treatment changes profoundly as adolescence progresses [6, 9, 10]; during the care transition, parents need to navigate between two opposing roles: while they need to take an active role, by coaching and supporting their child in becoming an independent and competent person, they also have to adapt to a less leading role and hand over the treatment responsibility to their child. Studies have found that parents of adolescents with chronic illness feel responsible for their child’s daily care and treatment and may fear handing over treatment responsibilities to their child [11, 12]. Parents ask for support to withdraw and adapt to their new role as ‘consultants’, instead of ‘managers’ [13–17]. Additionally, studies have found that parents are both anxious about leaving pediatric care and have an increased risk of developing anxiety and stress during their child’s transition [6, 10, 14, 18–20].

In the last decade, there has been an increased focus on transition programs targeting adolescents [21, 22]. A systematic review from 2017, a cohort study from 2018, and several guidelines conclude that health care professionals (HCP) also should focus on supporting parents in their transition and give parents help to support their child’s transition [5, 6, 23, 24]. Despite this, there is a lack of transition programs targeted to parents.

In conclusion, there is a need to develop interventions tailored to the needs of parents of adolescents with chronic illness, to support and prepare them for their child’s transfer to adult care. Thus, the aim of this paper was to describe the development, through participatory design (PD), of a comprehensive transfer program targeted to parents of adolescents with chronic illness.

## Overall methods

### Design

We chose a complex intervention design, because the final intervention will involve several interacting components and stakeholders and the intervention are developed and evaluated in multiple outpatient clinics across pediatric and adult care and implemented by multidisciplinary HCPs in a clinical setting. The study is based on the UK Medical Research Council’s (MRC) framework on developing, evaluating, and implementing complex interventions. The MRC framework consists of four key phases: development, piloting, evaluation, and implementation [25, 26]. This article will report the development phase.

**Fig. 1:**
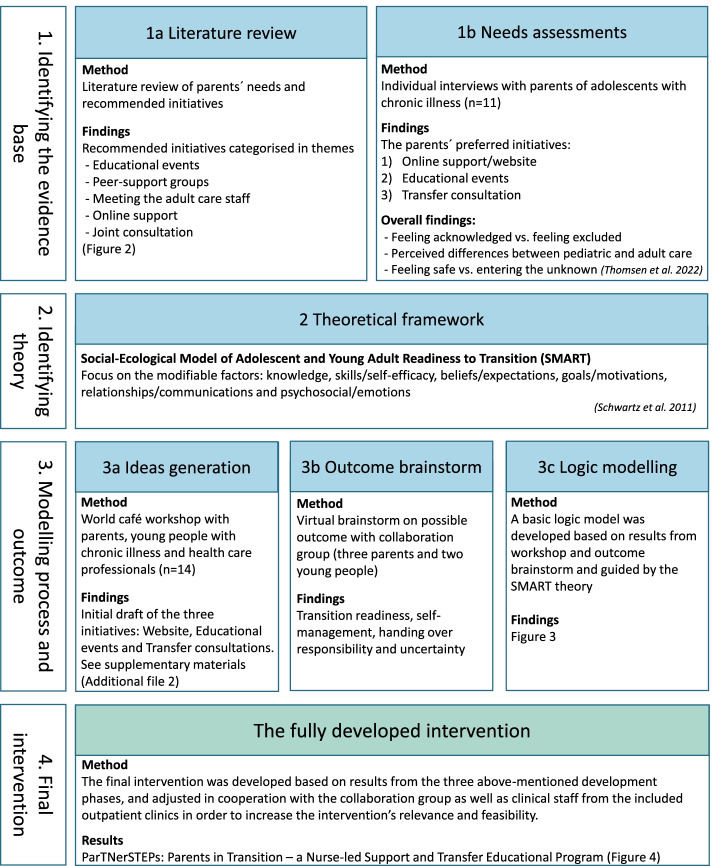
The overall process of developing a comprehensive transfer program

In addition to the complex intervention design, we chose PD to ensure that the users’ needs were represented and addressed in the program [27, 28]. The two first PD steps, “needs assessment” and “ideas generation”, were integrated into the development phase (see Fig. 1). By involving a collaboration group that included parents, young people, and HCPs, we will ensure the feasibility and relevance of the intervention in clinical practice and increase the chances of successful implementation [29].

### Setting

The intervention was developed at the pediatric and adult nephrology, hepatology, neurology and rheumatology outpatient clinics at Copenhagen University Hospital Rigshospitalet, Denmark. The eight clinics have teams with different mix of HCP backgrounds, working procedures and are affiliated at different locations and hospitals with different management. We chose these four specialties because low treatment adherence and non-attendance in their patient groups could result in permanent disabilities or critical consequences, e.g., organ failure, brain damage or permanent joint destruction.

In Denmark, adolescents transfer to adult care when they turn 18, irrespective of the adolescent’s maturity or transitional readiness. The existing transitional care in pediatrics includes assessment of adolescent transition readiness and split-visit consultations, where HCPs (nurses and physicians) both spend time alone with the adolescent, and with the adolescent and parents together. Focus in the split-visit consultations is on the adolescents and their youth life with chronic illness. The outpatient clinics currently have no initiatives that target parents.

### The collaboration group

Parents, young people and HCPs with affiliation to one of the four included outpatient clinics were invited to participate in the development of the intervention, by contributing their experiences, views, and needs. Parents who participated in initial needs assessment interviews [17] were invited to assist in the development of the program by being part of the collaboration group. We also invited young people from the local youth panel (a group of young people aged 14–25 with a wide range of chronic diseases (*n* = 18), along with HCPs from the involved outpatient clinics in pediatric and adult care (*n* = 21). A total of six parents, three young people and six HCPs chose to participate in the collaboration group (Table 1).

**Table 1:** Participants in the collaboration group (*n* = 13)

Parent (*n* = 6)	Parent of (gender, age)	Outpatient clinic
Father	Female, 19	Nephrology
Father	Female, 18	Nephrology
Mother	Female, 19	Nephrology
Mother	Male, 20	Hepatology
Mother	Female, 18	Rheumatology
Mother	Female, 20	Rheumatology
**Young people (*n*** **= 3)**	**Age**	**Outpatient clinic**
Female	19	Nephrology
Female	21	Hepatology
Female	23	Hepatology
**Health care professional (*n*** **= 6)**	**Affiliation**	**Outpatient clinic**
Physician	Pediatric	Hepatology
Head nurse	Pediatric	Hepatology, Rheumatology Nephrology, Neurology
Head nurse	Adult	Hepatology
Assistant head nurse	Adult	Nephrology
Nurse	Pediatric	Rheumatology
Nurse	Pediatric	Hepatology

## Developing a complex intervention

### Overview of the intervention development process

According to MRC’s framework, a complex intervention is developed in three stages: 1) identifying the evidence base, 2) identifying/developing theory, and 3) modelling process and outcomes. Figure 1 shows how these stages were executed and the individual steps towards the final development of the comprehensive transfer program. The intervention was developed in an iterative process over a 36-month period. Feedback from the collaborative group and all eight outpatient clinics were incorporated in the development. To reduce the complexity of the description of the intervention, the overall stages in the MRC framework are presented below, together with the respective method and findings of each stage.

### MRC stage 1: identifying the evidence base

The development of a complex intervention requires a review of the existing evidence to inform all steps of the development process [30]. In line with this, we conducted both a literature review and needs assessment interviews with parents.

#### Literature review

**Fig. 2:**
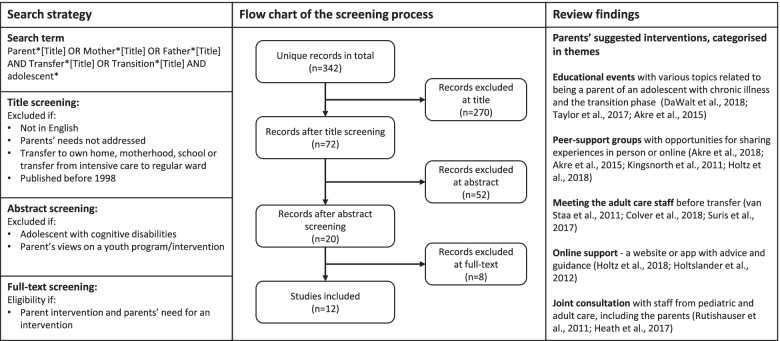
Search strategy, flowchart and results

Literature on parents’ needs and recommended interventions was identified through a literature search in the databases Medline and CINAHL (9th October 2018). The search strategy and findings are reported in Fig. 2. Extracts from the review formed the interview guide for the needs assessment interviews.

#### Needs assessment

In addition to the literature review, we conducted individual interviews with parents of adolescent with chronic illness in accordance with the PD first step (needs assessment). The principal aim in needs assessment is to identify and analyze specific needs by actively engaging the users [27].

A total of 11 parents of adolescents with chronic illness (aged 16–19) were interviewed during January and February 2019. After the interview, all parents were invited to assist in the development of the program, whereof six parents agreed to be a part of the collaboration group. A semi-structured interview guide was developed based on the results from the above-described literature review. It consisted of: 1) broad questions: to examine the parents’ feelings, experiences, or expectations in relation to their child’s transfer to adult care, and 2) structured questions: to identify preferred initiatives. Data were analyzed using the interpretive description method [31].

##### Findings

We found that the participants preferred initiatives that focused on the time up to transfer, including knowledge regarding adult care and getting to know the adult team. Our results pointed to three specific ideas for transfer initiatives, i.e., an informative website, educational events for parents, and a transfer consultation with HCPs from both pediatric and adult care. The overall findings can be found in Fig. 1. A full description of the method and findings is reported in detail elsewhere [17].

### MRC stage 2: identifying theory

Drawing on existing theory helps to identify what is essential, relevant and feasible, to inform the intended goals of the intervention and inform the content and delivery of any intervention [32].

#### Theoretical framework

We identified three transition theories, by Meleis [33], Schwartz et al. [8], and Geary and Schumacher [34]. They all state that the process of transitioning from pediatric to adult health care is part of a larger theoretical framework for transition affecting both young people and their parents [35]. We chose the transition theory Social-Ecological Model of Adolescent and Young Adult Readiness to Transition (SMART) developed by Schwartz et al. as the theoretical framework for the intervention [7]. The authors give the following as the definition; *SMART applies a social-ecological framework to transition readiness, emphasizing multiple factors, stakeholders, systems and their reciprocal relationships influencing the readiness for and likelihood of success in transfer to adult-oriented care* [8]*.* We found this theory relevant, given that our intervention is based on collaboration between multiple stakeholders (adolescents, parents and HCPs) and because it will take place in the context of a clinical setting and multidisciplinary teams. According to SMART, transition readiness is based on both pre-existing “objective” and less changeable factors (e.g., IQ, socio-demographics and medical status) and modifiable “subjective” factors in the patient, parents and HCPs that can be targeted by interventions. These factors are: knowledge, beliefs/expectations, goals/motivation, skills/self-efficacy, relationships/communication and psychosocial functioning/emotions [7]. The modifiable factors guided the modelling process (stage 3b).

### MRC stage 3: modelling process and outcome

Modelling a complex intervention can clarify the assumptions about the effect behind the intervention and increase the understanding of the mechanisms and components [36].

We chose to divide the modelling process into three substages: 1) Conducting a workshop with the collaboration group (ideas generation), 2) Online brainstorm regarding possible outcomes, and 3) Modelling the intervention using logic modelling.

#### Ideas generation

We conducted the workshop in line with the world café method, as this method engages people in meaningful group dialogue on complex issues, where all participants are the experts of their own lived experiences [37]. Young people with chronic illness, parents and HCPs from the collaboration group were invited to participate in the workshop.

The workshop took place January 2020 in an informal setting, in accordance with the world café principles described by Estacio and Toni, 2016 [38]. The world café concept was effectuated by having three tables focusing on one of the three preferred initiatives from the need assessments interviews (website, educational events and transfer consultations). The participants (*n* = 14) were divided into groups of four or five, so every group included at least one young person, one parent and HCP from different outpatient clinics. The groups rotated between each table every 20 min; thus, all participants debated all three intervention components. A facilitator at each table led the discussion with different questions in each round; 1) reflection questions, 2) content questions, and 3) design questions, respectively [see Additional file 1]. Prior to the table discussions, initial findings from the needs assessment interviews and modifiable factors from SMART was presented and discussed with the participants, in accordance with the principles of ideas generation (the second PD phase) [27]. The workshop was audio recorded and subsequently transcribed verbatim. Data (digital records and notes from participants and three table facilitators) were analyzed separately, table by table, using a thematic analysis approach inspired by Braun and Clarke’s 6-step model [39].

##### Findings

The analysis provided us with an initial draft of the three intervention components (website, educational events and transfer consultation). An overview of themes, categories and quotes can be found in Additional file 2. The main findings can be summarized as follows:

The participants wanted an informative website with a focus on knowledge sharing, with regard to not only expert knowledge but also personal experiences and advice on how to handle the transition process.

*“ … that at the same time there is this personal aspect, some personal stories with young people or parents of young people. Because I think the combination, where there is some knowledge and then something about how it is experienced to be this person.” (young person)*

The website should also contain an introduction to the adult department and give the parents the chance to interact with both medical experts and other parents.

Educational events should include several short presentations with various topics, such as patients’ rights and support opportunities, together with knowledge about adolescence and chronic illness. It was also important to the participants that parents and adolescents had the opportunity to ask questions and network with peers.

*“So it is not necessarily the educational evening in the teaching sense or meaning. It is much more that there is an opportunity to share some experiences and ask if there is anyone who has some good advice and how is it going with that” (HCP)*

The participants emphasized that a transfer consultation should focus both on coming to an official closure of the years of treatment in the pediatric department, and on an introduction to adult care.

*“I think it's important to be finished with the old. I think that is very important for the new process. So a good ending” (Parent)*

The transfer consultation should also focus on alignment of expectations, an introduction of the adolescent and her/his perceived need for parental involvement. Finally, the consultations should promote cooperation between departments and ensure a consistent and joint treatment plan.

*“Our mutual collaboration with children and adults becomes hugely important there, and so we have to air our dirty laundry internally and not [in front of the young person and the parents]” (HCP)*

#### Outcome brainstorm

We held two online brainstorm sessions with three parents and two young people from the collaboration group (April 2020). Parents and young people were asked to consider how a comprehensive transfer program targeting parents could affect them and their child/parents, respectively, and which changes they thought could happen. Parents’ and young people’s suggestions were drawn in a joint virtual mind map during the sessions.

##### Findings

The participants suggested a range of possible outcomes [see Additional file 3]. Suggested outcomes, such as transition readiness, self-management, handing over responsibility and uncertainty guided the final selection of outcomes.

#### Logic modelling

A logic model of the intervention can provide an overview of how an intervention works and how the theory and assumptions underlie the intervention. Thus, the logic model links short and long-term outcomes with the intervention’s activities and theory, by covering six core elements: Objectives, Inputs, Activities, Outputs, Outcomes and Impact [40, 41]. *Objectives* clarify the work focus, which usually describes changes in, e.g., knowledge and skills. *Inputs* cover the resources needed to implement the intervention (e.g., equipment, staff and cost). *Activities* describe specific intervention actions. *Outputs* cover the measurable product of program activities. *Outcomes* describe the intermediate outcomes, such as changes in, e.g., knowledge, skills or behavior. *Impact* covers the outcomes that partly depends on the interactions that lies outside of the control of the program [41].

**Fig. 3:**
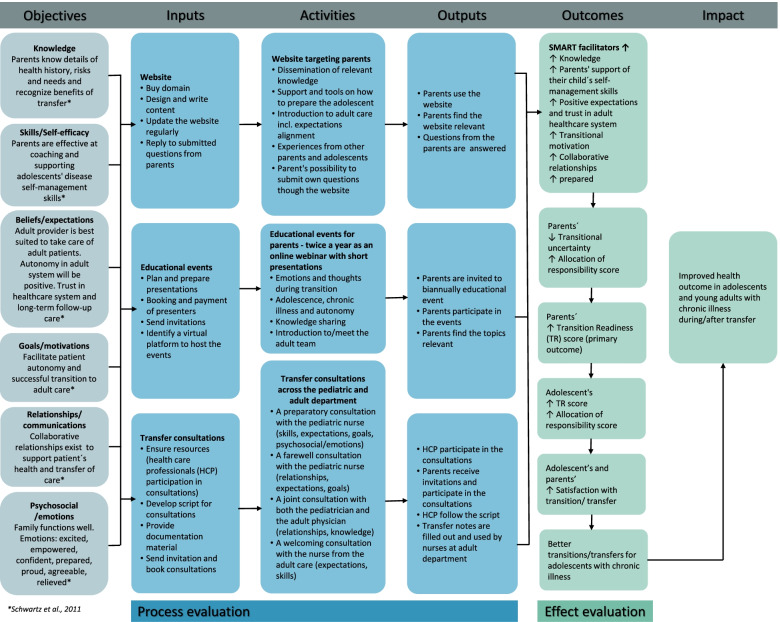
Basic logic model of the intervention

Authors ELT, KB, BAE, HH and SH all contributed to the final modelling of the intervention. The final model incorporated results from the workshop, outcome brainstorm and feedback from the collaboration group (Fig. 3). Facilitators of the transition process from the SMART theory guided the framework of the logic modelling process. The quality of the model was assessed by using the Kellogg Foundation checklist [40]. The model was presented and debated with the collaboration group before final approval.

### Final intervention

**Fig. 4:**
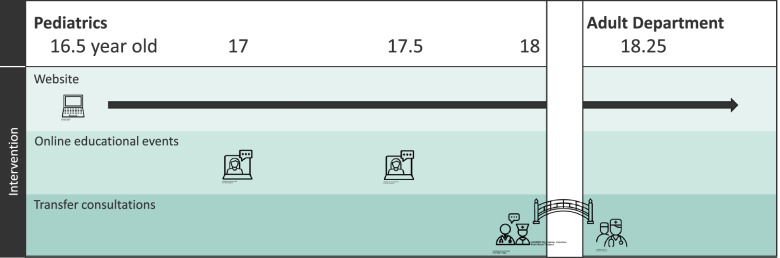
Model of ParTNerSTEPs

The final intervention, ParTNerSTEPs (Parents in Transition – a Nurse-led Support and Transfer Educational Program), was developed by the research team (authors) based on the logic model and results from the workshop and in close cooperation with the collaboration group (Fig. 4). ParTNerSTEPs consists of three components: 1) an informative website, where parents, adolescent and HCPs will provide content to the website and feedback on the website design and composition. 2) Educational events, which, because of the COVID-19 pandemic will be converted into online webinars. Time and weekday, among other things, will be decided by the parents. 3) Transfer consultations, where number of consultations, scripts and documentations tool will be developed in close cooperation with HCPs from all eight outpatient clinics (nurses and physicians from both pediatric and adult care).

#### Website

The website will be divided into six subpages, covering a range of needs and topics:

- *‘What is good to know before my child turns 18?’* focuses on relevant knowledge regarding social benefits, educational support and health care-related legal changes, e.g., confidentiality.
- *‘How can I prepare myself and my child during the transition?’* contains tools and advice on what the parents can expect of their adolescent child and how they can guide and prepare them.
- *‘What can I and my child expect of the adult department?’* gives a written and visual introduction to the four adult departments.
- *‘What do other parents and adolescents say about the transition?’* presents personal stories with experiences and advice. The site also links to relevant podcast, books and short videos regarding adolescence and chronic illness.
- *‘Questions and answers’* contain frequently asked questions and the opportunity for parents to submit their own questions, which will be answered and subsequently uploaded to the website.
- *‘Ideas for website improvements’.*

#### Online educational events

Online educational events will be offered to parents twice a year as a webinar with short presentations (10–15 min) on different topics. The webinar will take place on a weekday from 7 to 9 pm. Parents will be encouraged to invite their adolescent child to the webinars. The topics will change from time to time but will always contain: 1) personal experiences/presentations from an adolescent or parent, 2) presentations from professionals outside health care, e.g., student counselor, psychologist or social worker, 3) general presentations on adolescent medicine, e.g., adolescence, chronic illness and autonomy. At each webinar, the participants will be divided into disease-specific breakout rooms, where they can meet representatives from the adult department and network with the other parents.

#### Transfer consultations

Transfer consultations consists of, in total, four individual consultations:

- A preparatory consultation (3–6 months before transfer) with the pediatric nurse where a transfer document will be completed in collaboration with the adolescent and the parents. The transfer document will focus on the adolescent’s life with a chronic condition, independence, need for parents’ support and expectations/emotions regarding the transfer.
- A farewell consultation with the pediatric nurse (0–3 months before transfer), where the family gets the opportunity to say proper goodbye to the pediatric care team and be informed about what to expect in adult care.
- A joint consultation (at transfer) where both the pediatrician and the adult physician are present. Focus will be on a common treatment plan, partnership and meeting the adult treatment team.
- A welcoming consultation with the nurse from the adult care (0–3 months after transfer), where the transfer document will be discussed, and the family will be welcomed to the adult department.

## Discussion

The aim of this paper was to describe the development, through participatory design, of a comprehensive transfer program targeted to parents of adolescents with chronic illness. In accordance with the MRC framework, we have described the development of a complex intervention integrating PD. The MRC framework was a feasible and systematic step-by-step approach in designing an intervention with several interacting components that is carried out in a setting that spans across specialties as well as pediatric and adult departments. Our choice to apply PD allowed us to invite both young people, parents and HCPs to participate in the development of the intervention and thereby tailor the program to their specific needs and working routine. We believe that this approach has strengthened the entire intervention, as the collaboration group contributed with perspectives beyond our own perspectives as researchers [42]. PD also contributed to ensuring that the needs of the parents were met in the program, and for this reason we believe that the risk of drop-outs will be relatively low when evaluating the program.

Our comprehensive transfer program consists of three components: a website, online educational events and transfer consultations. Findings from our workshop showed that parents and young people preferred an informative website focusing on the dissemination of expert knowledge, personal experiences and advice on how to handle the transition process. Holtslander et al. have identified similar needs and preferences of parents and service providers of adolescents with type 1 diabetes, during the development of an online support intervention. They found a need for information, e.g., expert knowledge, emotional support, transition-related questions and the opportunity to connect with peers [43]. Another study among young people with ADHD from 2019 also found that the availability and communication of information was an essential component of the transition process [44]. This suggest that parents’ needs are similar across diagnoses. During the development of the educational events, we found that the participants were focused on usual youth topics as well as personal experiences and advice from parents and young people who already had transferred to adult care. Previous educational interventions have, like ParTNerSTEPs, addressed topics that are not only related to illness and treatment, but also topics that affect out-of-hospital life, such as college planning and social/youth life [45, 46]. Joint consultations have been recommended as one of the cornerstones of successful transfer [4–6, 47]. We also found that joint consultations were important for both young people, parents and HCPs. In line with evidence, the opportunity to meet the adult treatment team in a safe environment and the importance of collaboration across departments was highlighted as a facilitator of the transition process by participants [5].

The NICE guideline, Transition from children’s to adults’ services for young people using health or social care services, highlights the importance for involving parents, sharing working documents, meeting the adult care team and informing the family about what to expect from adult services [24]. Our comprehensive transfer program meets these recommendations in at least one of the three components (website, educational events and transfer consultation). Based on findings from our interviews and workshop, we have also included the opportunity for parents to benefit from other families’ approach by sharing their experiences and good advice on the website or at educational events.

Research has shown that involvement of parents results in better transitions, which is why it is recommended to support parents during transition [5, 6, 23, 24]. We have not been able to identify similar interventions that might suggest what a transition program targeted to parents would look like. The next step is, then, to evaluate the effect of our comprehensive transfer program by conducting a randomised controlled trial study involving the four outpatient clinics, together with process evaluation.

### Strengths and limitations

We chose to develop a comprehensive transfer program in close cooperation with parents and young people, who contributed with a variety of perspectives. This approach ensures better representation of the users’ needs and prevents potential inconsistency between their preferences and the scientific focus of research [42]. One limitation is the under-representation of male participants in the collaboration group. All young people were women, only two of the six parents were men, and only one parent of a male adolescent participated in our collaboration group. The absence of male participants is a common limitation and reflects clinical practice, given that mothers most often are the primary caregivers to children and adolescents with chronic illness [11, 48]. A limitation may also be the underrepresentation of physicians as well as representees from the neurology field. We recognize that the different diagnoses have different manifestations and one of the three components of the intervention are consultations in the respective specialties. However, our intervention is designed to strengthen the parents’ readiness for transition, which in principle is not linked to the various diagnoses, but more the changing role of parents during adolescence. As the intervention is nurse-led, we believe that the underrepresentation of physicians is not a limitation per se. Furthermore, physicians from alle eight clinics approved the intervention. Furthermore, the process of going through the many steps in the MRC framework is time consuming: it took 3 years from the literature review (2018) to the final adjustments in mid-2021. However, the lengthy development phase is also a strength, as the program’s legitimacy is ensured, based on the empirical, theoretical and comprehensive knowledge base.

## Conclusions

Transitional care is a complex process, because it involves adolescents, their parents and HCPs and covers modifiable subjective components. Thus, the MRC Framework was successfully applied to develop a comprehensive transfer program targeting parents of adolescents with chronic illness.

By incorporating the principles of participatory design in the development phase, we ensured that both parents’ and adolescents’ needs were represented and met in the program.

## Supplementary Information

**Additional file 1.**

**Additional file 2.**

**Additional file 3.**

## Data Availability

The datasets supporting the conclusions of this article are included within the article and its additional files (Additional files 1–3).

## Abbreviations

HCP: Health Care Professionals
MRC: Medical Research Council
SMART: The Social-ecological Model of Adolescent and young adult Readiness to Transition
PC: Participatory Design
