# Supplementary material for: Development of a complex intervention to support parents of adolescents with chronic illness transferring from pediatrics to adult care (ParTNerSTEPs)
Source: BMC Health Serv Res. 2022 Apr 12;22:485. doi: 10.1186/s12913-022-07888-5 (PMC9002046; doi:10.1186/s12913-022-07888-5)
Supplement: Supplementary file 1 — Additional file 1. [file 12913_2022_7888_MOESM1_ESM.pdf]

**Supplementary table 1** World café questions

| | **Online Support /website (Table 1)** | **Educational events (Table 2)** | **Transfer consultations (Table 3)** |
|---|---|---|---|
| ***Reflection questions (Round 1)*** | What makes a website good? | What are your experiences of good/ successful teaching/ lectures? | What, for you, is a good transition? |
| ***Content questions (Round 2)*** | Which topic should the website address? | What do you / parents need to know (what should the content be)? | What is important to discuss/address during a joint consultation? |
| ***Design questions (Round 3)*** | How should we design the website? | How should we design and plan the educational events? | How should we design and plan the joint consultations? |
