# Supplementary material for: Development of a complex intervention to support parents of adolescents with chronic illness transferring from pediatrics to adult care (ParTNerSTEPs)
Source: BMC Health Serv Res. 2022 Apr 12;22:485. doi: 10.1186/s12913-022-07888-5 (PMC9002046; doi:10.1186/s12913-022-07888-5)
Supplement: Supplementary file 2 — Additional file 2. [file 12913_2022_7888_MOESM2_ESM.pdf]

**Supplementary table 2** Findings and quotations from workshop

| WEBSITE<br>Theme | Category | Quotations |
|---|---|---|
| **Knowledge sharing** | Expert knowledge | "I also think about having a website with some knowledge and general information and that maybe describes something factual or describes some research studies about being young and ill, and that we have experienced it, and there may be those people and those problems" (Young person)<br><br>"Some of the things I can never answer are, what is the difference between everything that has to do with a social worker when you are a child and when you are an adult. I simply cannot remember, and I think that is general for everyone " (HCP) |
| | Personal experiences | "It might be cool to get some young people to talk about their illness, 'that's how it has been for me', 'I had those problems' and 'then I found a solution'" (Parent)<br><br>”…that at the same time there is this personal aspect, some personal stories with young people or parents of young people. Because I think the combination, where there is some knowledge and then something about how it is experienced to be this person.” (Young person) |
| | Research results | "There must also be something about theory, i.e. some research. You don't have to read it, but there must be something for everyone, a broad taste, so just upload some good articles around it" (HCP)<br><br>"I also think that it might be good to have information about what is going on in trials in Denmark on that disease." (Parent) |
| | Advice and guidance | "I'm also thinking about a section for parents on how they can transfer full responsibility to the young people. I think there are a lot of parents who have a really hard time letting go" (Parent) |
| **Introduction to adult care** | Contact info and instructions | "I also think that you easily should be able to find the phone-in time for this department" (Young person)<br><br>"There is something that is always missing on a website and especially for hospitals, and that's how hard it is to find an address [...]. And often that's why you go to a website, because you just have to find that address or telephone number. " (Parent) |

| | | |
|---|---|---|
| | Visual presentation | "That there is updated information and photos of the staff, so that they are not always new people, but that they are familiar faces" (Parent)<br><br>"I would like to suggest that there are small video clips where some people talk about it. Quite short and informative, where you see a real person" (Parent) |
| **Interactive** | Peer-to-peer support | "[...] some opportunity to find each other, I think. Because we hear that up on the wards, 'Oh, I would love to meet someone who has a child in the same [situation], who has the same"(HCP)<br><br>"I'm thinking a bit, in relation to what you are saying now, contact persons for parents. That you as parents who are in that situation can think, what do I do here? What will I benefit from? I just think it should be someone who is a little further along in the process than where you are, so you get confirmation that everything will be ok." (Parent) |
| | FAQ | "I sort of think, now at least I know the places where I've been where you can read overall questions that might be asked a lot - where you might be able to click on it and get answers." (Young person) |
| | Ask an expert | "The one about finances when you turn 18. I also had that. There could be a link to someone like a social worker and maybe even someone you could ask. Put questions to a social worker" (Parent) |
| **Tools** | Assessment form | "I think the one with a readiness checklist for the parents is really good. If you can make 10 points for the young person, then you can probably also do it for the parents." (HCP)<br><br>"Maybe one could do such a [readiness barometer], my child is 16 years old and then it should be possible to have control of half of it or 40% for the child. I think it will also be a good one if you can write 40% for the child and 60% for the parents and, then, if the parents think they are at 70%, then they can read that they might have to think about the fact that the young person her/himself needs to think about the disease "(Young person) |
| | Transition to autonomy – what to expect? | "Yes, that's also what makes it so hard as a parent to know when I can expect to do such a thing, and when is it ok to do such and such, and when can I expect him to take responsibility and do such and such" (Parent) |

| EDUCATIONAL EVENTS<br>Theme | Category | Quotations |
|---|---|---|
| **Content** | Knowledge about adolescence and chronic illness | "I think it's really important that parents get some knowledge about what it's like to be young and having a chronic condition [...], then you can probably, I think, use some more knowledge about what they are battling with, and the kind of challenges that they face, from some professionals. Because my experience is, where I'm coming from, that there are sometimes clashes between the parents and the young person. So if one can inform the parents, but with all due respect for the fact that of course they know their child and all that, but in what we really know and studies show and that the young people need" (HCP)<br><br>"Yes, but there is also the fact that it is difficult to be young and sick at the same time. It is difficult enough to be young and healthy and then be sick at the same time." (Parent)<br><br>"Well, it could be, my daughter has been extremely preoccupied with what this means for her. And we are also extremely preoccupied with it, can she have children? Is it a good idea? Is it hereditary? [ ...] precautions when traveling, what can you do? Then it has also been in connection with alcohol [...] So it has been a lot, the fact of managing your illness and an acceptance that this here is not going away. So I do not get a pill and then it's over in three months " (Parent) |
| | Supporting autonomy | "Prepare the young person to become an adult, that is, to take responsibility for their own lives. So mum is not always there to interpret what the doctors say. They have to, sort of, listen up themselves, and ask about things themselves, and take responsibility for things happening, because they should not expect nurses and doctors to just do the task" (Parent) |
| | Rights and support opportunities | "Then it should be something practical, I mean, help with how, as a social worker or something, who advised something about help with education" (Parent)<br><br>"Yes, and if you can not work, then you can get [help]. Yes, it will also be an idea [to have something about] rights in the education system?" (Parent) |
| | Transfer | "And there could be many different topics on how the transition is experienced, or what the differences are between being in a pediatric and adult ward." (HCP) |

| | | |
|---|---|---|
| | Networking with peers | "So it is not necessarily the educational evening in the teaching sense or meaning. It is much more that there is an opportunity to share some experiences and ask if there is anyone who has some good advice and how is it going with that" (HCP)<br><br>"... what gives you so much is that you meet someone else in the same situation. And you get so much, I have got so much out of it, both in the youth panel, where it's that, meeting other young people who are sick, and at patient associations help you to meet other people who have the same disease. It just gives so much. And you can feel that there are some people who can help me with some things, and I have also experienced that I have been able to help some people from my experiences. It's just so important that one does not forget it. " (Young person) |
| | Peer presenters | "I think it could also be nice that you also set the content so that you have different groups represented, so that there are also parents who come and teach parents, [...] that you can share the experiences among parents who have been in the situation and can pass on some knowledge "(Parent) |
| **The format** | Short presentations for discussion | "Well, for me, it has been about hearing a presentation and then having the opportunity to debate it afterwards [...] Both what is easy and what is difficult, what you have understood and what you have not understood and all that, things you have questions about because you had just not thought about that angle yourself" (Parent) |
| | Time for questions and reflection | "What I think when I stand on the other side, what I think, at least when I get the biggest feeling, is when they go home and it has been good, is when there has been time for reflection and to talk. And it requires an open and nice environment that invites you to talk and that there is no-one who give a dose. [...] If people need to spend time meeting physically, it must be because they get something other than what they can read on the website." (HCP)<br><br>"And that one can be allowed to ask questions. There will be a lot, what if and if it can be. So ask questions." (Parent)<br><br>"Then I also think that it is important to be able to ask, i.e. also about what you have talked about, that there will be time to ask, no matter what the content is. So something about being able to ask those who know" (Parent)<br><br>"I sat and thought, in the same overall way, that if you have to have a registration a month before, then there is also the chance to ask questions, so if there are some who have the same questions, then it is something that the psychologist can take up and say how it is generally" (Parent) |

| | | |
|---|---|---|
| | Including adolescents | "I think there should also be education for the young people, because it is they who must learn to commit themselves to this system when they have the prospect of being sick for the rest of their lives and it is not super easy" (Parent) |

| TRANSFER CONSULTATION Theme | Category | Quotations |
|---|---|---|
| **Parting** | Mental preparation | "So be prepared that now this here is ending, and in good time. So not just one consultation before, but in general, that you prepare mentally that now something new must happen and that's ok." (Parent) |
| | A proper ending | "I think it's important to be finished with the old. I think that is very important for the new process. So a good ending" (Parent) |
| | Talking positively about the new phase | "I also meet young people who are looking forward to coming to adult services and getting away from the clowns and getting away, and in that I just think it is important that we keep them in that positive thinking [...] So we should always support that, and if there are some who are happy, then they should just be allowed to be so" (HCP) |
| **Introduction to the new department** | Where to go | "... something so simple as being told exactly where to go. [...] Also quite specifically, because I think it will alleviate a bit of the unrest that is going on, because when you come to the same department, then it is well known and you know that you just have to stick your head in, if the door is closed. " (Parent) |
| | Introduction to differences | "And then also the fact that you are well and thoroughly introduced to the differences that there are [...] there are some things where it is like just expected that I know that you have to hand over urine sample the day before instead of when you come. And you cannot expect to know that when you have not been told" (Young person) |
| | Welcoming | "It's that, about having a clear welcome. To be very clear and vocal about it. 'Hi, you are new and we know that, we will help you find your way around' [...] I think that is very important." (Parent)<br><br>"... that you get a welcome [...] so you do not just feel like you are being let go somewhere and picked up somewhere else. So you get a more fluid transition." (Young person) |

| | | |
|---|---|---|
| | Alignment of expectations | "Yes, one thing is a clear expectations alignment, I mean, what I can expect to encounter." (Parent)<br><br>"And I also think to get to know what the purpose is. So when my daughter started at the adult ward, then we had an initial conversation, but it was not the doctor that she had been attached to [...], but we never really found out who she was, the doctor, was she attached at all, what was the purpose of the conversation? "(Parent) |
| **Introduction to the young person** | Getting to know the young person | "now I said it before, that you have to have an introduction to the adult ward, but also that the adult ward gets an introduction to the patient […] So at least in the handover conversation that you are allowed, so they are also aware that they know that we have a young lady who goes to high school or has a sabbatical year and we should probably know that, when we organize a treatment or we should probably just keep in mind, or it is probably someone who would like to go and travel for half a year, and how do we get it right. Because I just think it's important, because you just feel more like the organ you lack, than like a real human being, when you sit down." (Young person) |
| | Family dynamics | "... there has been a life with some illness and some experiences, there may have been both ups and downs in between, it can then be really relevant to know on the new ward. So you might know why parents do it that way or the young person does it because it's some habitual actions, and you're worried and nervous when you start at a new place." (HCP) |
| | Independence vs parental involvement | "I thought about the fact that some children are much more independent than others and some like to have mum and dad involved, but then there are also some who are more independent, and so I think that there might be an opportunity, at these meetings, to clarify what one's parents can be involved in. I want them to be involved in this and I can do that myself." (Parent) |
| | Summary of the illness trajectory | "but I think it has been nice to make a short summary of the entire course of the disease. Too often you have known the child since s/he was 16 years old or since s/he was 2 years old, so like saying that this is what we have been through and this is what we have done. [...] and that is also why I think it is quite important that the doctor who comes from adult services is also the doctor that they will see, the next time they come. So you kind of think you've passed the ball properly. "(HCP)<br><br>"And that you get to talk and have handed over and got to say that this young man has had a very short illness course, but it has been very severe, or it has been mild, so there hasn''t really been the big things. So you kind of know, that here we may have to take things steady, or here we can just get going with it, because she is this type and she takes the ups and down, and others have been through some other things, which makes it very difficult and it may need to be dosed differently. Differences are guaranteed." (HCP) |

| | | |
|---|---|---|
| **Shared understanding** | Understanding and acknowledging the parents´ role | "Perhaps the adult wards in general need to show an understanding of what it means for the young person to have to go from one ward to another, so that they do not talk down to them or talk down to the parents, such as, why do the parents want to come with you at all, like: 'tut, tut, have you taken your mother with you? I mean, you should not say that to such a young person" (Parent) |
| | Collaboration between departments | "I also think it's really important that pediatricians and adult services doctors talk together about what kind of treatment it is and that the pediatricians say, well we have done this and that. Because I experienced that I came to the adult ward and I did not get a consultation at all, I was just told when the next appointment would be, so you show up there and there, and then I showed up and then the doctor said, 'can you tell me what medicine you take' and then I said I take this and that and 'why do you take that?' you were asked, where you sort of, it is just not as nice, as a patient, to have the responsibility to have to, almost as an accusation. So that about talking well together and that there is a clear plan for this patient and that they kind of agree so that the patient does not stand and" (Young person)<br><br>"Our mutual collaboration with children and adults becomes hugely important there, and so we have to air our dirty laundry internally and not [in front of the young person and the parents]" (HCP) |
| **Creating security** | A named worker | "Well, I get the idea that you have to have some key people on this [...] that there simply have to be some who are key people in receiving the young people and who have a special interest and who will try to spend a little time and energy to get acquainted with how it is." (HCP) |
| | A known face | "I really like the idea of a nurse coming down [...] Because we all know how it is to walk into a strange place, we're looking for a familiar face that makes us feel safe, so to speak. So if you, like, have greeted someone at a totally strange place - that in itself makes it seem safer" (Parent) |
