# Supplementary material for: Development of a complex intervention to support parents of adolescents with chronic illness transferring from pediatrics to adult care (ParTNerSTEPs)
Source: BMC Health Serv Res. 2022 Apr 12;22:485. doi: 10.1186/s12913-022-07888-5 (PMC9002046; doi:10.1186/s12913-022-07888-5)
Supplement: Supplementary file 3 — Additional file 3. [file 12913_2022_7888_MOESM3_ESM.pdf]

Supplementary table 3 Outcome of brainstorm session

Interviews with three parents (two mothers and one father) and two adolescents (both transferred to adult care) – about their thoughts on the potential impact of the intervention in the adolescent and the parents:

	Potential parental outcomes	Potential adolescent outcomes
**Adolescents'** suggestions regarding outcome measurements	- Increased feeling of **peace of mind** and **at ease** when their child transfer to adult care<br>- Better **prepared**<br>- Increased **knowledge** on rights and support possibilities<br>- Increased **overview** of rights and support possibility<br>- Feeling **less alone**<br>- Better **prepared to support** their child in the transition process	- Increased feeling of **continuity**<br>- Increased feeling of **peace of mind**<br>- Increased **overview** over e.g., rights<br>- Increased knowledge in order to **take on the responsibility**<br>- Better **communication** between the parents and the adolescent<br>- Increased feeling of **being understood** by their parents<br>- **Fewer conflicts** between the parents and the adolescents<br>- Feeling that the parents are **less worried**
**Parents'** suggestions regarding outcome measurements	- Increased **knowledge** on rights and support possibilities<br>- Parents will **start** to prepare and talk about the **transition earlier**<br>- Increased confidence in **handing over the responsibility** to the adolescent<br>- Better **prepared**<br>- Better **understanding** for the adolescent's life<br>- **Less problematic** transfer<br>- Increased feeling of **safe/confidence /peace of mind**<br>- Use **VAS** as a measuring tool, e.g., How **confident** are you in letting your child take her/his medicine by her/himself?<br>- A better **ending** at pediatrics<br>- Experiences of better **patient care** with continuity<br>- Which initiative was **most useful** and why?	- Increased **knowledge** of rights and support possibilities<br>- Better **equipped for** transfer<br>- Increased **self-management**<br>- Increased feeling of **acknowledged** by the health care professionals<br>- Increased feeling of **safe/confidence /peace of mind**<br>- Improved **well-being**<br>- A more **holistic consultation** with a focus on the whole youth-life<br>- Increased feeling of being **understood**<br>- Less **lonely**
